# Chromosome-scale genome assembly of an important medicinal plant honeysuckle

**DOI:** 10.1038/s41597-022-01385-4

**Published:** 2022-05-24

**Authors:** Hang Yu, Kun Guo, Kunlong Lai, Muhammad Ali Shah, Zijian Xu, Na Cui, Haifeng Wang

**Affiliations:** 1 State Key Laboratory for Conservation and Utilization of Subtropical Agro-Bioresources, Guangxi Key Lab for Sugarcane Biology, College of Agriculture, Guangxi University, Nanning, 530005 China; 2 Institute of Medicinal Plant Development, Chinese Academy of Medical Sciences, Peking Union Medical College, Beijing, 100193 China; 3 Beijing Huigene Biotechnology Co., Ltd, Beijing, 100070 China

**Keywords:** Comparative genomics, Genome informatics

## Abstract

*Lonicera japonica* (honeysuckle) is one of the most important medicinal plants and widely utilized in traditional Chinese medicine. At present, there are many varieties of honeysuckle used in cultivation, among which Sijihua variety are widely cultivated due to its wide adaptability, stress resistance, early flowering and high yield. In this study, we assembled the genome of Sijihua, which was approximately 886.04 Mb in size with a scaffold N50 of 79.5 Mb. 93.28% of the total assembled sequences were anchored to 9 pseudo-chromosomes by using PacBio long reads and Hi-C sequencing data. We predicted 39,320 protein-coding genes and 92.87% of them could be annotated in NR, GO, KOG, KEGG and other databases. In addition, we identified 644 tRNAs, 2,156 rRNAs, 109 miRNAs and 5,502 pseudogenes from the genome. The chromosome-scale genome of Sijihua will be a significant resource for understanding the genetic basis of high stress-resistance, which will facilitate further study of the genetic diversity and accelerate the genetic improvement and breeding of *L. japonica*.

## Abstract (summary)

**Table:** 

Measurement(s)	Lonicera japonica • RNA sequencing • genome assembly • sequence annotation
Technology Type(s)	SMRT Sequencing • RNA sequencing • Hi-C • biomolecular annotation design
Factor Type(s)	Genotype
Sample Characteristic - Organism	Lonicera japonica
Sample Characteristic - Environment	occurrence
Sample Characteristic - Location	Shandong Province

## Background & Summary

*Lonicera japonica* (Caprifoliaceae), is perennial and evergreen twining vine, commonly known as Jinyinhua or Rendong, plays a very important role in traditional Chinese medicine^1^. Meanwhile, *L. japonica* has been cultivated as an ornamental plant in many areas because of its varying colors and attractive smell^2^. *L. japonica* firstly recorded as medicine can be traced back to the Jin dynasty in China, then it also recorded in ‘Ben Cao Gang Mu’ which is famous classical book of Chinese material medica^1^. The first place in China where *L. japonica* was planted massively is Fengqiu county of Henan province, and it has been used to treat exogenous wind-heat, febrile disease, sore, carbuncle, furuncle and some infectious diseases^3^. It was found that the extracts of *L. japonica* and its chemical components have a variety of pharmacological effects, including anti-inflammatory, antibacterial, antiviral, antioxidant, liver protection and anti-tumor^1^. As *L. japonica* being used and cultivated in more and more areas, its chemical constituents have been studied widely. *L. japonica* contains a variety of medicinal ingredients, such as flavonoids, essential oil, triterpenoid soap and organic acids^4,5^, which allows honeysuckle to perform many pharmacological functions.

*L. japonica* has always been the object of researches and many scientists mainly focused on the biosynthesis of active medicinal ingredients and differential gene expression patterns between different tissues with the help of transcriptomics^6–8^. However, with the development of sequencing technology and the reduction of sequencing cost, progressively plant genomes are available, including *L. japonica*, whose Institute of Medicinal Plant Development (IMPLAD) germplasm registration number is 10107428^9^. The study about Lj10107428 provided proof for a whole genome duplication (WGD) event and showed the expression of related biosynthetic genes was correlated with the accumulation of carotenoids and suggested the role of carotenoid degradation in the dynamic coloring of *L. japonica* by assembling the whole genome and transcriptomic analysis^9^. Nevertheless, a genome of one variety is not enough to represent the genetic resources of the species. The availability of the genomes of different varieties can help to improve the genetic resources of the species and understand the reasons for the trait alternations between different varieties. Therefore, we also sequenced and *de novo* assembled genome of a *L. japonica* variety that is called ‘Sijihua’, which is largely planted in Pingyi county, Shandong province. It’s reported that Sijihua are the most cold-resistant variety by studying different varieties of *L. japonica* in different regions^10^.

Here, we generated a chromosome-scale of the genome assembly of the variety of Sijihua using the combination of PacBio long reads, Illumina reads and the Hi-C sequencing data. Approximately 886.04 Mb genome was assembled with the contig N50 length of 1.58 Mb. A total of 826.50 Mb (93.28%) of the assembled sequences were anchored to 9 pseudo-chromosomes (Table 1). We predicted 39,320 protein-coding genes, and 92.87% of each gene were assigned by BLASTP against NR, GO, KOG, KEGG and other databases. We identified 644 tRNAs, 2,156 rRNAs, 109 miRNAs and 5,502 pseudogenes (Table 2). We also identified 255,264 simple sequence repeats (SSRs), and 40,252 are polymorphic SSRs (Table 3). The genome assembly of variety Sijihua is a valuable material to the germplasm resources of *L. japonica*, and helps researchers to explore the specificity of different varieties. The results also provide valuable clues to the molecular basis of cold-resistance traits of Sijihua and will facilitate further genetic improvements.

**Table 1:** Genome assembly and assessment of Sijihua and Lj10107428 genomes.

Assembly		Sijihua	Lj10107428
Genome-sequencing depth (X)	PacBio sequencing	98.88	90(ONT)
	Illumina sequencing	61.48	56.91
	Hi-C	103.65	94.86
Estimated genome size (Mb)		817.45	887.15
Estimated heterozygosity (%)		0.74	1.27
Number of scaffolds		967	145
Total length of scaffolds (bp)		886,131,823	903,813,648
Scaffolds N50 (bp)		79,566,881	84,431,753
Longest scaffold (bp)		116,908,140	125,163,164
Number of contigs (bp)		1,519	919
Total length of contigs (bp)		886,040,423	903,735,777
Contigs N50 (bp)		1,578,755	2,148,893
Longest contig (bp)		12,449,837	19,544,413
GC content (%)		34.32	43.5
Mapping with Illumina reads (%)		99.75	NA
CEGMA assessment (%)		95.85	NA
Completeness BUSCOs (%)		97.03	97
Complete single-copy BUSCOs (%)		91.33	92.6
Complete duplicated BUSCOs (%)		5.70	4.4

**Table 2:** Genome annotation of Sijihua and Lj10107428 genomes.

Annotation	Sijihua	Lj10107428
Number of predicted protein-coding genes	39,320	33,961
Average gene length (bp)	4,640	3,527
Average exon length (bp)	1,480	1,118
Average exon number per gene	4.87	4.63
Average intron length (bp)	3,160	2,407
miRNAs	109	33
rRNAs	2,156	138
tRNAs	644	104
Percentage of repeat sequence (%)	64.76	58.21
Copia (%)	15.93	8.98
Gypsy (%)	19.14	13.77
LINE (%)	2.61	2.33
SINE (%)	0.34	0.17
DNA transposons (%)	5.82	7.67
Pseudogenes	5,502	18
Percentage of Functional annotation genes	92.87	NA

**Table 3:** SSRs annotation of Sijihua and Lj10107428 genomes.

SSR type/species	Sijihua	Lj10107428	polySSRs
Di-nucleotide	192,362	144,713	34,140
Tri-nucleotide	54,009	31,248	5,135
Tetra-nucleotide	6,395	4,580	654
Penta-nucleotide	1,526	1,100	181
Hexa-nucleotide	972	566	142
Total	255,264	182,207	40,252

## Methods

### Sample collection, library construction and genome size estimation

**Fig. 1:**
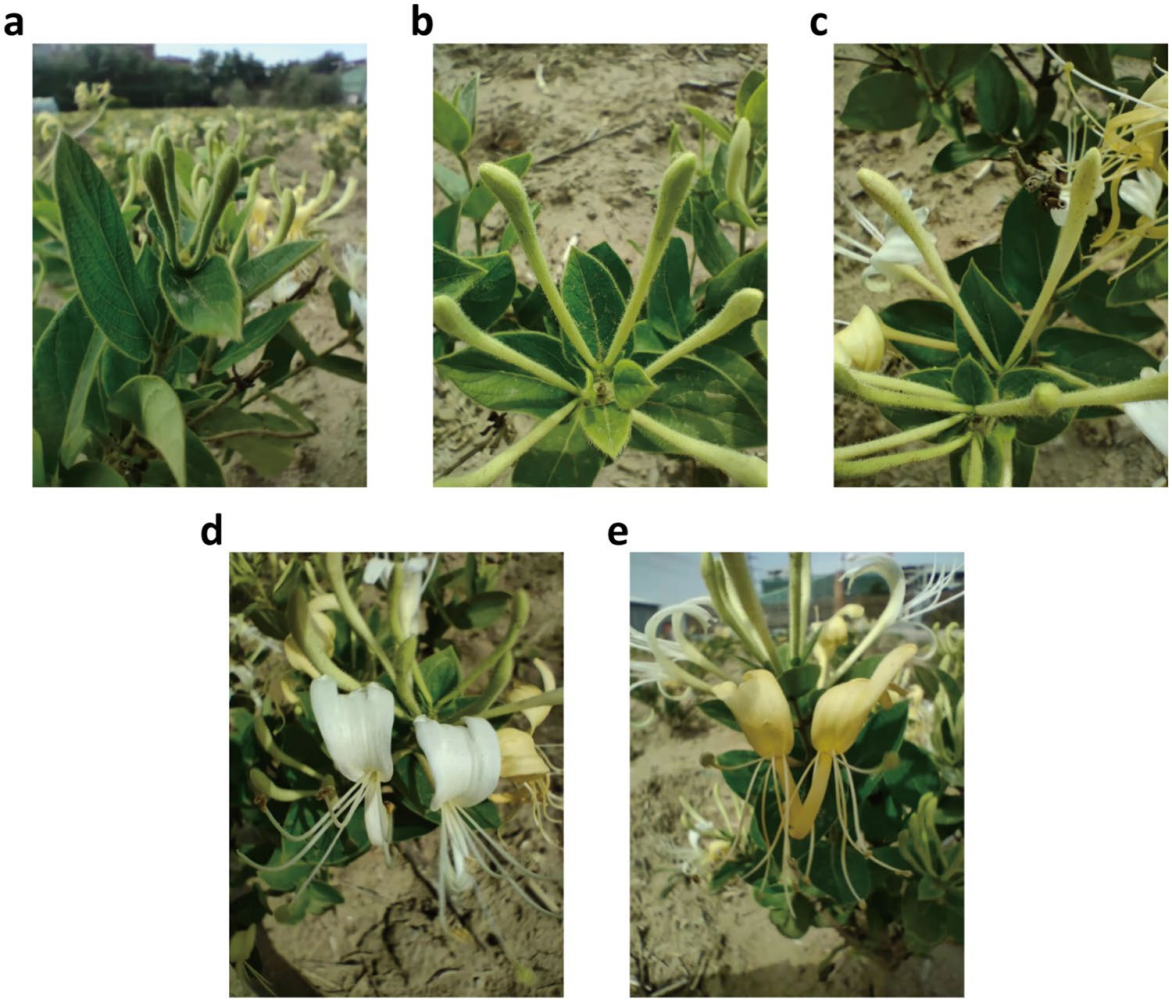
The five growth stages of honeysuckle. (**a**) The juvenile bud stage. (**b**) The third green stage. (**c**) The complete white stage. (**d**) The silver flowering stage. (**e**) The gold flowering stage.

High-quality genomic DNA was extracted from young-fresh leaf tissue of Sijihua using CTAB (cetyl trimethylammonium bromide) method, and the samples were collected from the Zhongke Honeysuckle Planting Cooperative in Pingyi County, Shandong, China (Fig. 1). The qualified genomic DNA was broken to the target fragment (350 bp) by ultrasonic shock and the Illumina library was constructed through end repairing, adding 3’ A tail, ligating adapters and enriching with PCR. The fragment size and quality of the library were detected by 2100 and Q-PCR. Next, the sequencing of the library were performed by using Illumina Novaseq 6000, which finally generated 31.48 million reads, 50.26 Gb of raw data, which covered 61.48 × of the genome. PacBio library were constructed by using BulePippin to screen the target fragment that was interrupted by g-tube, subsequently was sequenced with PacBio Sequel II system. Consequently, the two SMRT-cells generated a total of 4,461,375 reads with N50 size of 29,096 bp. Totally we got 87.61 Gb sequencing data, accounting for 98.88 × of the entire genome. Fresh leaf tissue of honeysuckle was used to construct a library for Hi-C analysis. The fresh tissue was fixed with formaldehyde, attaining interacting loci to be bound to one another, and then cross-linked DNA was digested by restriction enzyme Hind III. Sticky ends were labeled with biotin during its repairing. Next, the interacting DNA fragments were ligated, purified and finally broke them into 300 bp ~ 700 bp fragments. Each ligated DNA fragment was marked with biotin and streptavidin beads were used to pull-down the interacting DNA fragments to complete the Hi-C library construction. The libraries were then sequenced on Illumina Novaseq 6000 platform, which generated 307,357,239 pairs of reads and 91.84 Gb clean data, which cover 103.65x of the genome.

**Fig. 2:**
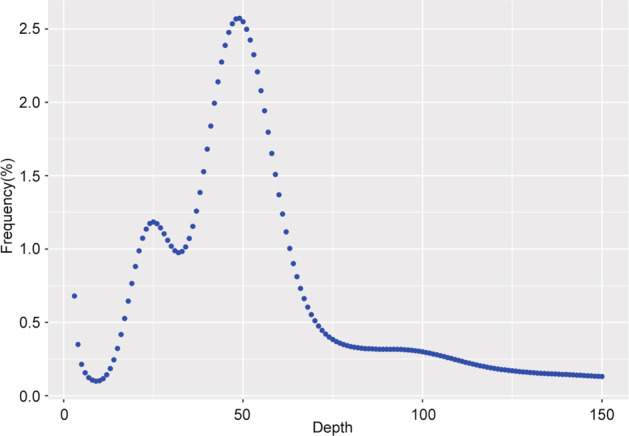
19-kmer distribution in the honeysuckle genome.

A k-mer (k = 19) analysis was constructed using 61.48 × Illumina data to estimate the genome size, proportion of repeat sequence and heterozygosity^11^. From the 19-kmers distribution, we could estimate the heterozygosity and repeat ratio of the Sijihua genome to be 0.74% and 51.8%, respectively and the estimated genome size was 817.45 Mb (Fig. 2 and Table 1).

### RNA sequencing and analysis

Total RNA was extracted by using RNeasy Plus Mini Kit (Qiagen) from different development stages, including juvenile bud (JB), green bud (GB), white bud (WB), silver flower (SF), and golden flower (GF). Libraries were constructed by TruSeq RNA Library Prep Kit v.2 (Illumina, San Diego, CA, USA). Each library was constructed with three biological replicates, and 150 bp paired-end reads were sequenced by using the Illumina NovaSeq 6000 platform. Raw reads were trimmed by using TRIMMOMATIC (v.0.39)^12^, and the clean reads were aligned to the reference genome by HISAT2 (v. 2.2.1)^13^ with default parameters, and only uniquely mapped reads were kept. Expression value was estimated by StringTie (v. 2.1.5)^14^ as FPKM (Fragments per kilobase of exon model per million reads mapped). Genes with FPKM > 0.5 were considered as expressed, and used for further analysis. Differentially expressed genes (DEGs) were identified by DESeq2 (v 1.28.1)^15^ with default parameters. RNA-seq data of LJ10107428 were downloaded from National Genomics Data Center (https://ngdc.cncb.ac.cn) with BioProject ID (PRJCA001719).

### *De novo* genome assembly

PacBio long reads were error corrected using Canu v1.5^16^ and the top 40 x coverage of the longest corrected reads were subsequently assembled by SMARTdenovo^17^. To improve the accuracy of the assembly, the 61.48x Illumina short reads used in the genome survey were applied to three rounds of correction by Pilon v1.22^18^. The high-quality Hi-C reads were used to cluster, order and orient the contigs onto pseudo-chromosomes by using the LACHESIS^19^. We preliminary assembled the PacBio long reads into contig sequences of 886.04 Mb, including 1,159 contigs with N50 of 1.58 Mb, and the longest contig is 12.45 Mb. These contigs were further anchored onto 9 pseudo-chromosomes, accounting for 93.28% of the assembled genome. The final chromosome-scale genome assembly of Sijihua was 886.13 Mb with a scaffold N50 of 79.57 Mb (Table 1).

### Repeat annotation

*De novo* and structure-based predictions were integrated to annotate repetitive sequences. LTR_FINDER v1.05^20^ and RepeatScout v1.05^21^ were primarily used to build a *de novo* repeat sequences library of Sijihua genome, which was classified by using PASTEClassifier v1.0^22^ and merged with Repbase v19.06^23^ database as the final repeat sequences database. Structure-based predictions were performed by using RepeatMasker v4.05^24^ based on the constructed repeat sequences database. We identified 573.89 Mb (64.76%) of repetitive sequences in Sijihua genome. Most of these repeat sequences are Class I (53.57%) retrotransposons, including *Copia, Gypsy, LINE* and *SINE*, accounted for 15.93%, 19.14%, 2.61% and 0.34% of the entire genome, respectively. In addition, Class II DNA transposons make up 5.82% of the genome (Table 2).

### Protein-coding genes prediction and other annotations of the genome

Prediction of protein-coding genes was based on *ab initio* gene predictions, homology-based predictions and transcriptome-based predictions. *Ab initio* predictions were performed by Genscan v3.1^25^, Augustus v3.1^26^, GlimmerHMM v1.2^27^ v3.0.4, GeneID^28^ v1.4, and SNAP (version 2006–07–28)^29^. For homology-based prediction, GeMoMa v1.3.1^30^ was used to annotate the gene models in Sijihua using amino acid sequences from *Daucus carota*, *Helianthus annuus*, *Lactuca sativa*, *Lonicera japonica* and *Arabidopsis thaliana* genome. For RNA-seq-based prediction, the clean RNA-seq reads were aligned to the assembled genome using Hisat^31^ v2.0.4 and Stringtie^32^ v1.2.3, and then TransDecoder^33^ v2.0, GenemarkS-T^34^ v5.1 and PASA^35^ v2.0.2 were jointly used for final coding-gene prediction. Finally, 39,320 gene models were predicted after integrating results of the three methods of predictions by EVidenceModeler^36^ v1.1.1 (Table 2). For non-coding RNAs annotation, microRNA and rRNA were detected by aligning the assembled genome against the to Rfam^37^ database using BLASTN. tRNA was identified by tRNAscan-SE^38^. Finally, we totally identified 2,909 non-coding RNAs, including 109 miRNAs, 2,156 rRNAs and 644 tRNAs.

The sequence of pseudogenes is similar to that of functional genes, but whose original function is lost due to insertions, deletions and other variants. The predicted protein sequences were used to search for homologous gene sequences on the genome through BLAT^39^ alignment, and then GeneWise^39^ was used to search for immature stop codons and frameshift mutations in gene sequences. In total, 5,502 pseudogenes were predicted (Table 2).

For gene functional annotation, we aligned the predicted protein-coding gene sequences against public functional databases using BLAST^40^ v2.2.31 (-evalue 1E-5), such as NR^41^, KOG^42^, GO^43^, KEGG^44^ and TrEMBL^45^. In addition, the motif and domain information were annotated using InterProScan^46^ through searching against public databases, including PROSITE^47^, HAMAP^48^, Pfam^49^, PRINTS^50^, ProDom^51^, SMART^52^, TIGRFAMs^53^, PIRSF^54^, SUPERFAMILY^55^, CATH-Gene3D^56^ and PANTHER^57^. As a result, more than 92% of protein-coding genes were annotated, and 1,376 conserved motifs and 36,282 domains were identified.

### Identification of SSRs and polymorphism

Reference genomes of Sijihua and Lj10107428 were analyzed for the identification of simple sequence repeats (SSRs) and polymorphism by using CandiSSR^58^ with default parameters. We identified 255,264 and 182,207 SSRs in Sijihua and Lj10107428 genomes, respectively. Moreover, we found 40,252 SSRs are polymorphic between Sijihua and Lj10107428 genomes (Table 3). In addition, among all the SSRs identified in Sijihua genome, the most abundant SSRs motifis are di-nucleotide (192,362), followed by tri-nucleotide (54,009), tetra-nucleotide (6,395), penta-nucleotide (1,526), and hexa-nucleotide (972).

### Global genome comparison of the Lj10107428 and Sijihua

**Fig. 3:**
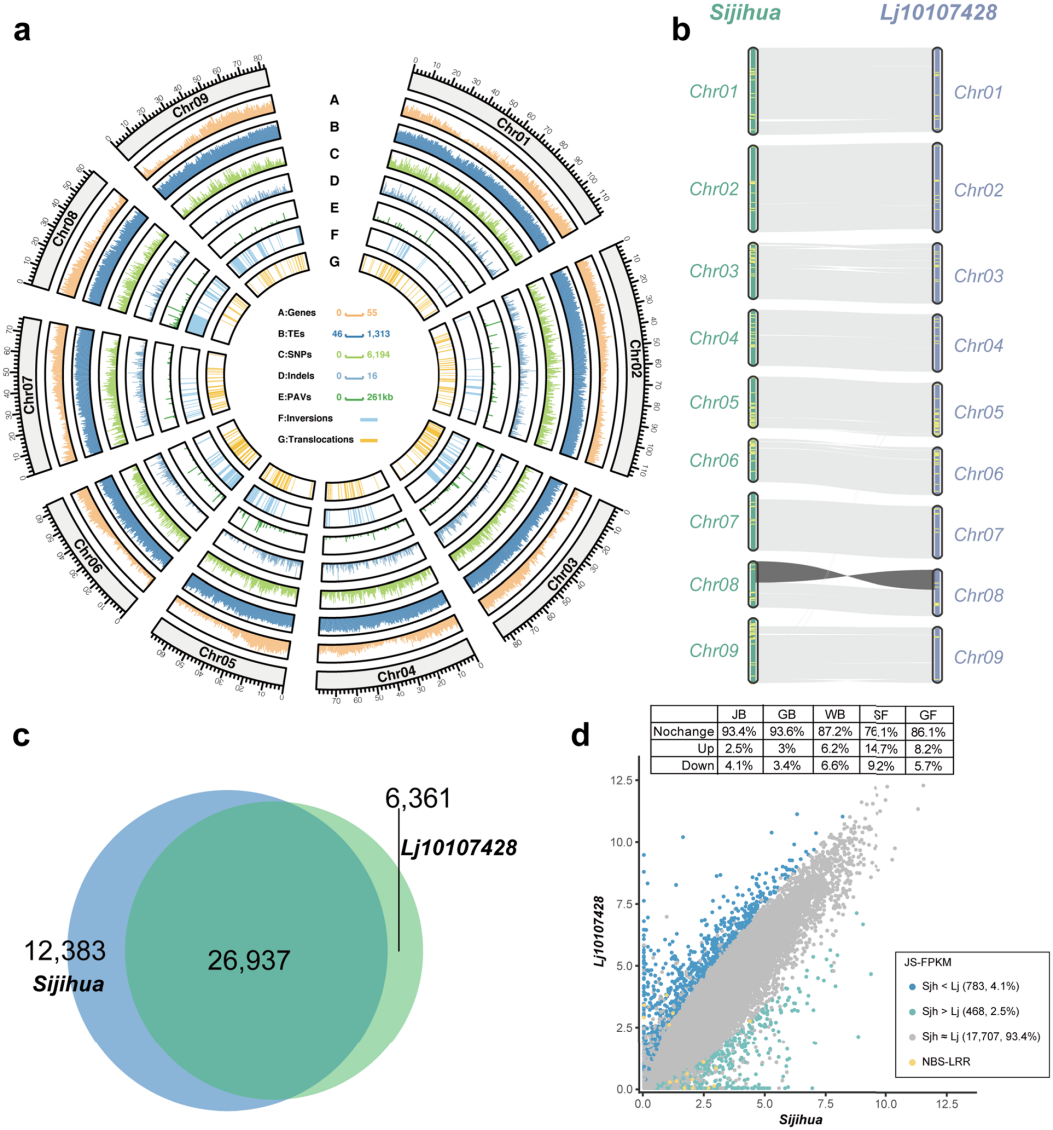
Comparative genomic analysis between Sijihua and Lj10107428 varieties of honeysuckle. (**a**) Genomic features landscape of the Sijihua genome. Density of genes, TEs, SNPs, indels, PAVs, inversions and translocations were calculated in a 500 Kb sliding window. (**b**) Gene collinearity between Sijihua and Lj10107428 varieties. NBS-LRR genes were annotated as yellow dot across genome. (**c**) Venn diagram of the overlapped genes between Sijihua and Lj10107428 genomes. (**d**) Expression level comparison of shared genes in Sijihua and Lj10107428 varieties.

Genome comparison between Sijihua and Lj10107428 was performed by using the NUCmer program embedded in MUMmer4 with parameters –mum –l 40 –c 100, then the delta alignment file was filtered by delta-filter with parameters -1, and finally show-snps was used to identify single nucleotide polymorphisms (SNPs) with parameters -ClrT. SyRI v1.5^59^ was used to extract structure variations (SVs) based on the alignment file with tab-delimited text format performed by show-coords program. MCScanX^60^ was used to identify gene collinearity, including within and between genomes. In total, we identified 2,996,015 SNPs between Sijihua and Lj10107428. Among them, 14.44% of the SNPs were located in the genic region. Comparison between Lj10107428 and Sijihua genomes, we found 5,150 small insertions/deletions (indels, length shorter than 500 bp), and more than 4.5 Mb of presence/absence variation (PAV, length longer than 500 bp). Notably, we identified 895 Sijihua-specific genomic sequences (2.61 Mb in total), and 418 Lj10107428-specific genomic sequence (1.84 Mb in total) longer than 500 bp. These PAV segments were unevenly distributed across the chromosomes, and the longest PAV sequence was a 261 Kb segment on chromosome 2 (Fig. 3a). In addition, we also found 156 inversions ( >1,000 bp) and several translocations ( >10 kb) between these two genomes. The genome sequence comparison between Lj10107428 and Sijihua reveals high collinearity. Although some structure variations were detected between these two genomes, they primarily consist of large syntenic block with high degrees of collinearity (Fig. 3b). In addition, we identified 301 large syntenic blocks between Sijihua and Lj10107428, containing 25,128 syntenic genes. Specificity, there is an inverted region of approximate 19 Mb on chromosome 8 between Sijihua and Lj10107428 genomes (Fig. 3b). We also noticed that 561 and 302 NBS-LRR genes located in to Sijihua and Lj10107428 genomes, respectively (Fig. 3b). By comparing the annotated protein-coding genes, we found that 26,937 genes of Sijihua and Lj10107428 were shared by reciprocal best hit of BLAST algorithm with parameter (E-value < 1e-10). More species-specific genes were found in Sijihua (12,383) than that in Lj10107428 (6,361) (Fig. 3c).

From the 18,958 overlapped expressed (FPKM > 0.5) genes between Sijihua and Lj10107428, most of them (>93.4%) did not show differential expression variation at juvenile bud stage. However, hundreds of genes were differential expressed, including 16 NBS-LRR genes. Notably, 11 of 16 NBS-LRR genes were highly expressed in Sijihua at juvenile bud stage. This observation is consistent across other flower development stages (Fig. 3d).

## Data Records

The raw data of PacBio, Illumina and Hi-C sequencing were submitted to the National Center for Biotechnology Information (NCBI) SRA database with accession number SRP353698^61^ under BioProject accession number PRJNA794868. RNA-seq data were deposited into the NCBI (accession number PRJNA813701)^62^. The assembled genome had been deposited at GenBank with accession number SAMN24662184^63^. In addition, the genome annotation file had been submitted at the Figshare^64^.

## Technical Validation

### Evaluation of the genome assembly

To evaluate the quality of genome assembly, bwa (version: 0.7.10-R789; mode: aln) was used to align the Illumine short reads with the reference genome, and 99.75% of the Illumina short reads were mapped to the reference genome CEGMA^65^ v2.5 was used to assess the integrity of the final genome assembly. The CEGMA database contained 458 conserved core eukaryotic genes, while our assembled genome contained 439 (95.85%), which suggested that our assembled genome contains most of the core eukaryotic genes. BUSCO^66^ v4.0 was used to assess the integrity of our genome assembly by using the Embryophyta database of OrthoDB v10. Of the 1614 expected embryophyta genes, our genome contains 1,556 (97.03%). Together, these three evaluation systems demonstrate the high integrity of our assembled genome (Table 1).

**Fig. 4:**
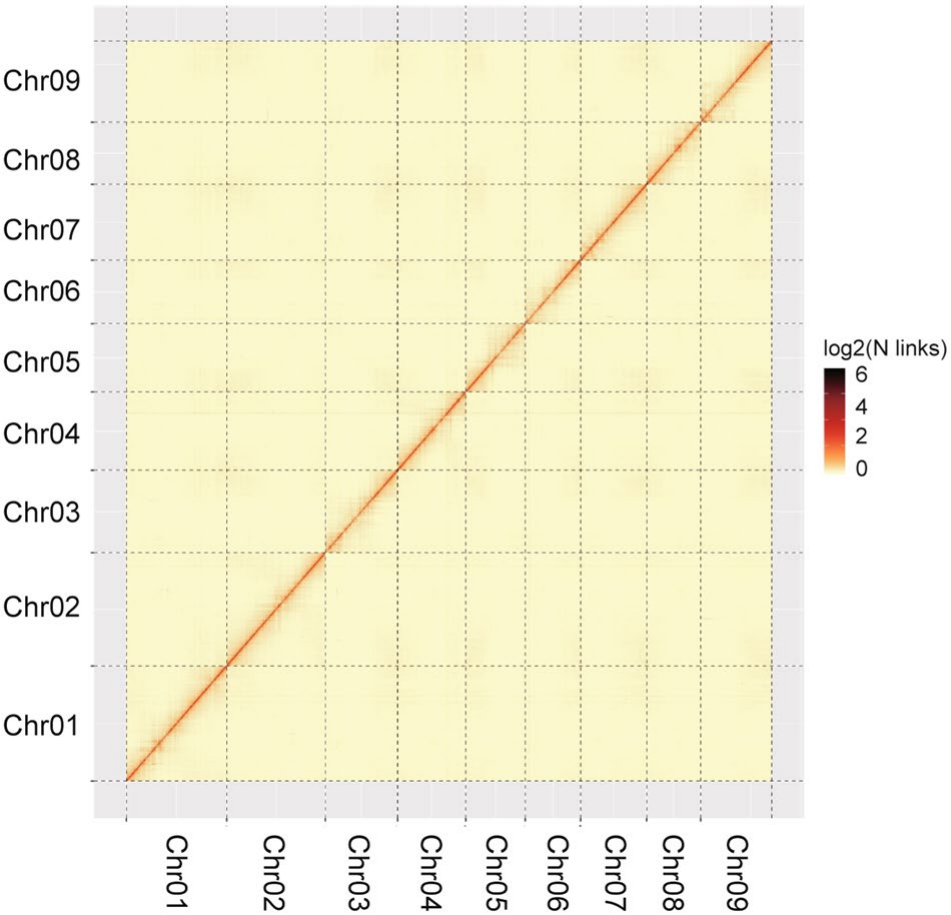
Hi-C contact map of the chromosome-scale assembly of Sijihua. Hi-C interaction matrix shows the pairwise correlations among 9 pseudomolecules. The intensity of the dark color is scaled to the strength of the correlation.

Furthermore, to assess the result of Hi-C assembly, and the number of Hi-C read pairs coverage between any two bins acts as a strength signal of interaction between the two bins. As chromosomal interaction heatmap shown, within each group, it was found that the intensity of interaction at the diagonal position was higher than that at the non-diagonal position (Fig. 4), which was consistent with the principle of Hi-C-assisted genome assembly and proved that the genome assembly was accurate.

## Data Availability

BUSCO: --evalue 1e-03, -sp Arabidopsis.LACHESIS:CLUSTER_MIN_RE_SITES = 30;CLUSTER_MAX_LINK_DENSITY = 2;CLUSTER_NONINFORMATIVE_RATIO = 2;ORDER_MIN_N_RES_IN_TRUN = 49;ORDER_MIN_N_RES_IN_SHREDS = 49.Software parameters of repeat annotation: default parameters for LTR_FINDER, RepeatScout, and PASTEClassifier. RepeatMasker: -nolow -no_is -norna -engine wublast.Software parameters of gene prediction: default parameters for Genscan, Augustus,GlimmerHMM, GeneID, SNAP, GeMoMa, Stringtie, TransDecoder, GeneMarkS-T, and EVM.Hisat:--max-Intronlen 20000, --min-intronlen 20. PASA: -align_tools gmap, -maxintronlen20000.GenBlastA: -e 1e-5.BLASTP: -e 1e-10.CandiSSR: perl CandiSSR.pl -i crl.file -o out_path/.Default parameters were used in other software unless otherwise specified.
